# A new approach to measure interocular suppression in amblyopia and strabismus

**DOI:** 10.1016/j.mex.2023.102527

**Published:** 2023-12-18

**Authors:** Chuan Hou

**Affiliations:** The Smith-Kettlewell Eye Research Institute, United States

**Keywords:** Amblyopia, Strabismus, Orientation discrimination, Interocular suppression, Visual perception, A new method to measure interocular suppression in amblyopia and strabismus

## Abstract

Interocular suppression is commonly estimated by the contrast “balance point” between the eyes in individuals with amblyopia, in which the depth of suppression is defined as the increased contrast in the amblyopic eye that is perceptually matched to the fellow eye. However, this method may not be suitable for individuals with strabismus who have normal or even better contrast sensitivity in the non-fixating eye. In this study, I introduced a new approach that can be used for measuring interocular suppression for both amblyopia and strabismus without balancing contrasts between the eyes. The method consists of the following components:

• The stimuli have equal contrasts in both eyes.
• The percepts of objects seen monocularly and binocularly are compared.
• Interocular suppression is defined as the percept of object in the amblyopic eye is suppressed when an object is presented in the fellow eye.

## Graphical abstract

**Figure:**
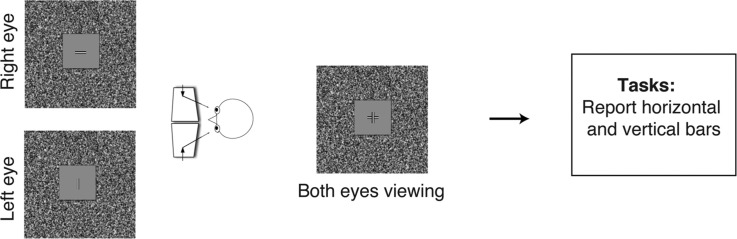

Specifications table

**Table:** 

Subject Area:	Neuroscience
More specific subject area:	Interocular suppression measurement in amblyopia and strabismus
Method name:	A new method to measure interocular suppression in amblyopia and strabismus
Name and reference of original method:	None
Resource availability:	The script in Matlab is available by request

## Method details

### Background

Amblyopia (‘lazy eye’) is development disorder of spatial vision and affects about 3–5 % of the population [1]. Chronic visual suppression to the visual input from the non-preferred eye is a key factor in developing amblyopia [2], as well as a critical consequence of preventing binocular confusion and diplopia in strabismus [2,3].

There is no gold standard to measure interocuar suppression in the clinic and the research laboratory. The depth of suppression in amblyopia is commonly evaluated as the amount by which the monocular contrast increment threshold for an eye was elevated by stimulation in the contralateral eye [4]. Using a similar concept, a contrast “balance point” between the eyes in individuals with amblyopia has been used as an index to estimate the depth of suppression [5,6] and also to evaluate binocular vision restoring by alleviating suppression in amblyopic training [7,8]. Importantly, this measurement is based on reduced contrast sensitivity in the amblyopic eye of amblyopia [9], which cannot be used in some strabismic individuals who have strong suppression but with normal or even better contrast sensitivity in the non-fixating eye [9]. In this study, I introduced a new approach that avoids contrast balance between the eyes and can be used for measuring interocular suppression in both amblyopia and strabismus, including some strabismic individuals who have visual suppression but with normal or even better contrast sensitivity in the non-fixating eye. We presented stimuli with equal contrasts in both eyes and compared the percept of objects seen monocularly and binocularly. Interocular suppression is defined as the percept of object in the amblyopic eye is suppressed when an object is presented in the fellow eye.

### Method design

Suppression within the context of binocular vision refers to an inhibitory influence of the fellow eye over the amblyopic eye when both eyes are viewing [5]. Therefore, our method design for estimating interocular suppression is: if any suppression exists in individuals with amblyopia or strabismus, the visual percept of the amblyopic eye or the non-fixating eye of strabismus viewed monocularly should be better than the percept viewed binocularly when an object simultaneously presenting to the other eye, represented by the *ratio* of binocular viewing over the monocular viewing *less than 1*.

**Fig. 1:**
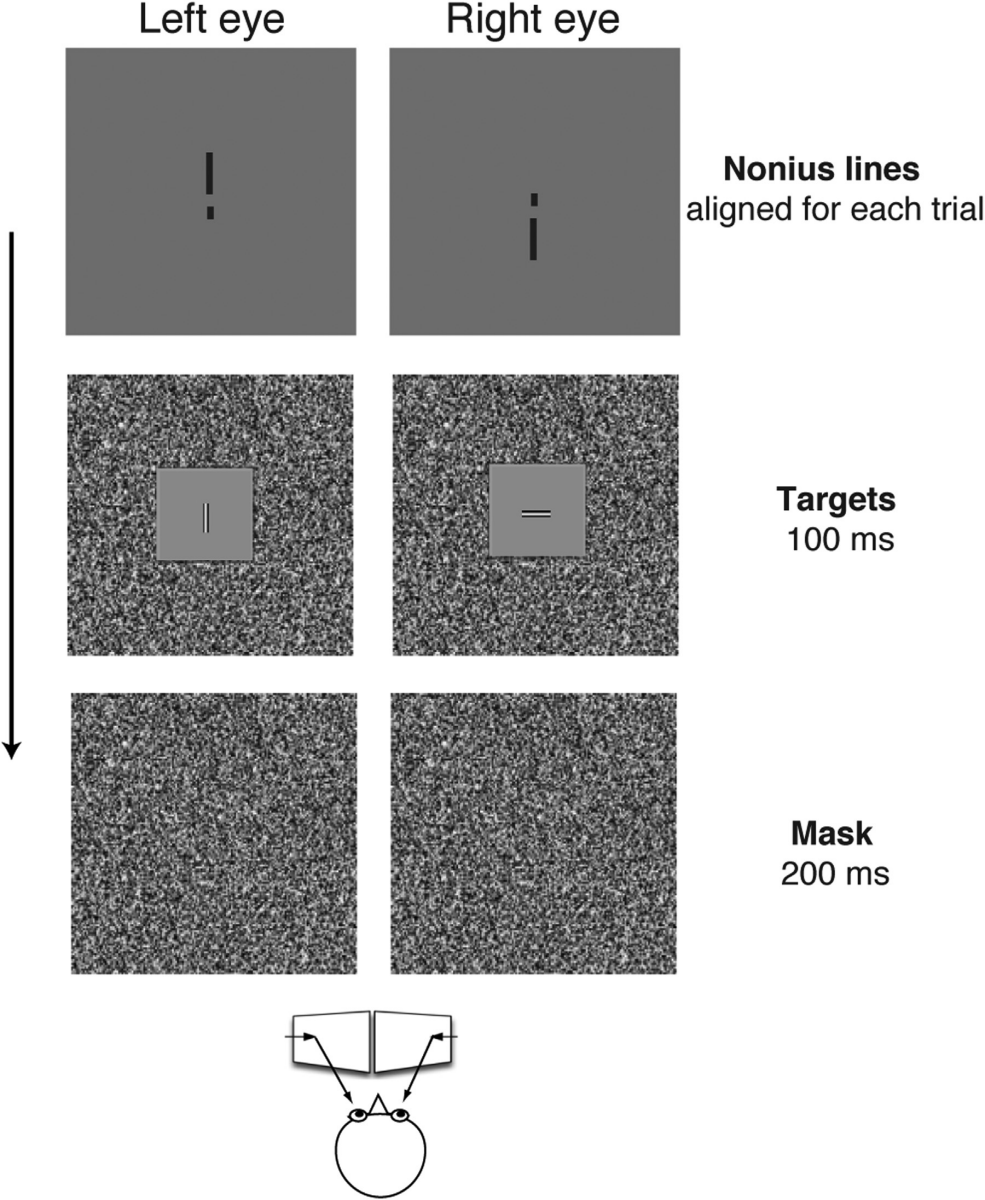
Illustration of method for measuring suppression in individuals with amblyopia and strabismus. For a given trial (shown in binocular presentation), two bars (vertical bar in the left eye and horizontal bar in the right eye) are presented for 100 ms. The bars are highly visible (above or at 35 % contrast with 1 cpd of spatial frequency) to ensure that the amblyopic eye can see the bar. The tasks are to report how many bars they saw (one or two) and to report the orientation(s) of the bar(s) by button press. The temporal sequence of a given trial as the following: A central fixation point and nonius lines appear prior to the initiation of all trials, to ensure that the mirror stereoscope remains properly aligned. Then, the observer initials the trial. The stimuli are presented for 100 ms followed by a 200 ms-noise mask. The trials are self-initiated and the observer is requested to respond as accurately as possible with no time limit and no feedback is given.

As shown in Fig. 1, the stimuli consist of highly visible vertical and horizontal bars presented to the different eyes. The purpose of using horizontal and vertical bars is to allow observers to identify the percept of the stimulus in each eye. However, the stimuli of bars are not critical, as any objects that can be identified with different eyes should do the work. Using the bars with low spatial frequency (1 cpd) and high contrast (35 %) in each eye is to allow the object could be clearly seen by the amblyopic eye with poor vision acuity. Unlike the traditional methods with either increasing stimulus contrast in the amblyopic eye or decreasing stimulus contrast in the fellow eye [5], [6], [7], [8], in our design, we remain the same stimulus contrasts in the two eyes. The stimuli are presented to two identical monitors with a dichoptic setting. In our validation study [10], the stimuli were presented on two identical cathode ray tube monitors (Sony, Tokyo, Japan) at a viewing distance of 86 cm. The stimuli should be viewed through mirror stereoscope, as so, both the horizontal and vertical deviations in individuals with amblyopia and/or strabismus can be adjustable by mirrors to align the nonius lines under the best optical correction. The stimulus presentation is 60 to 100 ms, which is short to avoid producing rivalry [11]. A 200 ms-noise mask following to the stimuli is to avoid afterimage effect. When experiment is initiated, the length of the bar is adjusted so that its orientation is identified correctly on or better than 75 % of the trials when presented monocularly to the amblyopic eye or the non-fixating eye of strabismus. This is a critical step, so that the length of the bar can be further used in the mixed binocular and monocular presentation stimuli. In each block of psychophysical procedures, half of the trials are binocular presentation stimuli (i.e., horizontal bar presents to one eye and vertical bar presents to the other eye simultaneously) with the horizontal bar randomly assigned to each eye. The other half of the trials are monocular presentation stimuli, in which only one bar (either horizontal or vertical) presents to either the left or the right eye with equal probability. For example, in the case of 100 trials in the block, 50 trials are binocular presentation and the other 50 trials are monocular presentation (25 trials with bar only in the left eye and other 25 trials with bar only in the right eye).

The tasks for observers are to report how many bars they saw (one or two) and, if they saw only one, to report its orientation by a button press, for example, press horizontal arrow to indicate seeing horizontal bar. If they saw both horizontal and vertical bars, the observers press two buttons to report orientations of the two bars, for example, press both horizontal arrow and vertical arrow to indicate seeing both horizontal and vertical bars. The binocular presentation trials measure suppression by determining whether the bar in the amblyopic eye is suppressed when a bar is presented in the fellow eye. Monocular presentation trials are included to measure monocular visibility in the amblyopic eye or the non-fixating eye of strabismus. On a given trial, the observer does not know whether only one bar is presented or whether two bars are presented. The correct percent in the amblyopic eye or the non-fixating eye of strabismus seen monocularly and binocularly will be calculated separately. The depth of suppression is defined as the ratio of binocular correct percent over monocular correct percent seen by the amblyopic eye or the non-fixating eye of strabismus (*suppression* = *binocular% / monocular%*), with a smaller number of ratios indicating a stronger suppression.

### Method validation

Using the method described in this study, we measured interocular suppression for 11 strabismic amblyopes, who showed significant correlation between the degree of suppression and the depth of amblyopia [10], indicating that the individuals with worse visual acuity had stronger suppression. This method was further validated in the use of measuring interocular suppression in adults with amblyopia and strabismus and monitoring reduction of suppression along with visual acuity improvement across amblyopic training sessions [12].

## Conclusions

In summary, a new approach for measuring interocular suppression in individuals with amblyopia and strabismus is introduced in this study. This method has been demonstrated having a significant correlation between the degree of suppression and the depth of amblyopia in two studies [10,12]. This method, rather than contrast “balance point” between the eyes, can be used in both amblyopia and strabismus that have either low or normal contrast sensitivity in their non-favorite eye.

## Ethics statements

The protocol of this method and validation studies [10,12] were approved by The Smith-Kettlewell Institutional Review Board and conformed to the tenets of the Declaration of Helsinki. Informed consent form was obtained from all the participants after the experimental procedures were explained in the validation studies [10,12].

## CRediT authorship contribution statement

**Chuan Hou:** Conceptualization, Methodology, Validation, Writing – review & editing.

## Declaration of Competing Interest

The author declares no competing financial interests or personal relationships that could have appeared to influence the work reported in this paper.

## Data Availability

No data was used for the research described in the article.
